# Hypothermia reduces VEGF-165 expression, but not osteogenic differentiation of human adipose stem cells under hypoxia

**DOI:** 10.1371/journal.pone.0171492

**Published:** 2017-02-06

**Authors:** Nick C. Leegwater, Astrid D. Bakker, Jolanda M. A. Hogervorst, Peter A. Nolte, Jenneke Klein-Nulend

**Affiliations:** 1 Department of Orthopaedics, Spaarne Hospital, Hoofddorp, The Netherlands; 2 Department of Oral Cell Biology, Academic Centre for Dentistry Amsterdam (ACTA), University of Amsterdam and VU University Amsterdam, MOVE Research Institute Amsterdam, Amsterdam, The Netherlands; University of California Davis, UNITED STATES

## Abstract

Cryotherapy is successfully used in the clinic to reduce pain and inflammation after musculoskeletal damage, and might prevent secondary tissue damage under the prevalent hypoxic conditions. Whether cryotherapy reduces mesenchymal stem cell (MSC) number and differentiation under hypoxic conditions, causing impaired callus formation is unknown. We aimed to determine whether hypothermia modulates proliferation, apoptosis, nitric oxide production, VEGF gene and protein expression, and osteogenic/chondrogenic differentiation of human MSCs *under hypoxia*. Human adipose MSCs were cultured under hypoxia (37°C, 1% O_2_), hypothermia and hypoxia (30°C, 1% O_2_), or control conditions (37°C, 20% O_2_). Total DNA, protein, nitric oxide production, alkaline phosphatase activity, gene expression, and VEGF protein concentration were measured up to day 8. Hypoxia enhanced *KI67* expression at day 4. The combination of hypothermia and hypoxia further enhanced *KI67* gene expression compared to hypoxia alone, but was unable to prevent the 1.2-fold reduction in DNA amount caused by hypoxia at day 4. Addition of hypothermia to hypoxic cells did not alter the effect of hypoxia alone on *BAX*-to-*BCL-2* ratio, alkaline phosphatase activity, gene expression of *SOX9*, *COL1*, or osteocalcin, or nitric oxide production. Hypothermia decreased the stimulating effect of hypoxia on *VEGF-165* gene expression by 6-fold at day 4 and by 2-fold at day 8. Hypothermia also decreased VEGF protein expression under hypoxia by 2.9-fold at day 8. In conclusion, hypothermia decreased *VEGF-165* gene and protein expression, but did not affect differentiation, or apoptosis of MSCs cultured under hypoxia. These *in vitro* results implicate that hypothermia treatment *in vivo*, applied to alleviate pain and inflammation, is not likely to harm early stages of callus formation.

## Introduction

Fractures are generally accompanied by soft tissue trauma that is aggravated by subsequent surgical stabilization. The interruption of arterial vascular flow causes regional ischaemia and hypoxia, resulting in inflammation [1]. Cryotherapy seems to be a modulator of the posttraumatic inflammatory reaction, but results obtained do not unanimously agree on the way it affects inflammation. Cryotherapy has been reported to reduce posttraumatic microvascular dysfunction, inflammation, and structural impairment in a rodent model [2]. However, hypothermia prolongs the inflammatory response systemically and locally in fracture hematomas in a porcine model [3]. Cryotherapy can be applied in the acute recovery phase of musculoskeletal trauma and after orthopaedic surgical interventions, such as knee arthroplasty to prevent pain and inflammation [4]. Currently, the clinical effect of cryotherapy is being investigated in postoperative hip fracture patients [5], even though the effect of application of cryotherapy on osteoblast precursor proliferation and differentiation during bone tissue repair has not been clearly established.

During the process of bone repair, the interruption of vascular flow activates the coagulation and formation of a fracture hematoma, which has a remarkable angiogenic capacity [1]. Moreover, hypoxia is a key factor in bone repair [1]. It increases human mesenchymal stem cell (MSC) migration rates and improves their tissue regenerative potential in a murine hind limb ischaemia model [6]. Furthermore hypoxia induces recruitment of fibroblasts and osteogenic progenitor cells via the production of reactive oxygen species (ROS) [7,8]. At *low* concentrations, ROS functions as a messenger to enhance wound healing [9]. It targets survival pathways such as mitogen-activated protein kinase (MAPK) and phosphoinositide 3-kinase (PI3K)/Akt [8,10]. ROS also targets hypoxia-inducible factor-1 (HIF-1), which upregulates gene expression of vascular endothelial growth factor (VEGF), an angiogenesis and vasculogenesis-inducing agent as well as a bone-metabolism cytokine that stimulates the differentiation and chemotactic migration of osteoblast precursor cells [11–13]. However, hypoxia is not always beneficial for bone repair. Physiological ROS formation is disrupted during ischaemia and subsequent reperfusion [14]. Pathological hypoxia sustained during ischaemia causes ROS accumulation [14], which has been implicated in secondary tissue damage. Cooling of ischaemic tissues using cryotherapy, which decreases muscle temperature to 23°C at the thigh of healthy individuals [15], might overcome some of the adverse effects of the pathological hypoxic state and excessive ROS formation. However, the effect of cryotherapy on cell metabolism in fracture haematomas is currently unknown.

Cryotherapy diminishes the cell’s metabolic rate of glucose, oxygen, and lactate production by 2 to 4-fold per 10°C reduction in the mammalian central nervous system [16]. Synovial lactate concentrations remain stable despite a decrease in blood flow (indicated by increased ethanol exchange ratio) when cryotherapy is applied in patients recovering from arthroscopy, suggesting a decrease in energy requirements [17]. A reduction in ROS concentration by hypothermia has been shown to attenuate the apoptotic cascade in murine nerve cells [18]. Thus, cryotherapy likely reduces cell metabolism in haematomas of fracture patients, thereby it might reduce harmful concentrations of ROS. In addition, hypothermia blocks ß-catenin degradation via the PI3K/Akt pathway in a focal ischaemic rat model, resulting in decreased cell injury and apoptosis [19], and activation of the PI3K/Akt pathway is suggested to enhance osteogenic differentiation of MSCs [20].

On the other hand, hypothermia reduces osteoblast proliferation and differentiation while promoting osteoclast function in cultured rat calvariae [21]. Taken together, these findings warrant further investigation of induced hypothermia effects on early stages of bone healing. To date no studies have addressed whether hypothermia affects osteogenic differentiation and proliferation of MSCs, be it in a positive or negative way, under hypoxic conditions.

We aimed to determine whether hypothermia modulates proliferation, apoptosis, nitric oxide (NO) production, VEGF gene and protein expression, and osteogenic/chondrogenic differentiation of human mesenchymal stem cells *under hypoxia*. We hypothesized that hypoxia stimulates osteogenic/chondrogenic differentiation, VEGF gene and protein expression, NO production, and apoptosis, and inhibits MSC proliferation, but that hypothermia attenuates these hypoxia-induced effects.

## Materials and methods

### Donors

hASCs were isolated from abdominal subcutaneous adipose tissue as waste material after abdominoplasty from six Caucasian healthy female donors (age 31–56) at Tergooi Hospital, Hilversum, The Netherlands. Our study has been conducted within the framework of the “Medical Research Involving Human Subjects Act (WMO) exemption” as ruled by the Medical Ethical Committee of the VU University Medical Centre, Amsterdam, The Netherlands (date: 17-03-2016; reference no: 2016.105). The use of all human materials in this study has been approved by the Medical Ethical Committee of the VU University Medical Centre (“Medisch Ethische Toetsingscommissie VU medisch centrum”; protocol number 2005/128) after obtaining written informed consent.

### hASCs isolation and culture

Human adipose tissue obtained by resection was stored in sterile phosphate buffered saline (PBS) at 4°C overnight and processed within 24 h, as described previously [22,23]. In brief, adipose tissue was minced, washed with PBS, and enzymatically digested with 0.1% collagenase A (Roche Diagnostics, Mannheim, Germany) in PBS containing 1% bovine serum albumin (BSA; Roche Diagnostics). The resulting cell pellet containing the hASCs was resuspended, and viability and cell number was measured with a NucleoCounter^®^ (NC-100^™^, ChemoMetec, Allerod, Denmark). Confirmation of stem cell phenotype has been confirmed earlier by surface marker expression [22,23]. The attached ASCs are virtually all positive for markers CD29 (cell adhesion marker), CD73, CD90, and CD105 (MSC-associated markers), CD166 and HLA-ABC (leucocyte surface markers), while they do not express leucocyte surface markers CD45 or HLA-DR [22,23]. For cell culture, single cell suspensions of cryopreserved hASCs were thawed and seeded at 4-12x10^4^ cells/cm^2^ in α-Modified Eagle’s Medium (α-MEM; Gibco, Paisley, UK) supplemented with 5% platelet lysate (PL; VU University Medical Centre, Amsterdam, The Netherlands), 0.2% (vol/vol) heparin (5,000 U/ml), and 1% antibiotic-antimycotic solution (10,000 U/ml penicillin (Gibco), 10 mg/ml streptomycin (Gibco), and 25 μg/ml amphotericin B (Sigma)), and cultured under 5% CO_2_ and 20% O_2_, at 37°C. Platelet lysate is known to induce osteogenic differentiation of hASCs [24,25]. Upon reaching 80–90% confluency, cells were harvested by incubation with 0.25% trypsin/0.1% ethylenediaminetetraacetic acid (EDTA; Gibco) in PBS for 5 min at 37°C. All cells used were from passage 4 or less. For experiments, hASCs were seeded at 10,000 cells/cm^2^ in 6-well dishes containing α-MEM supplemented with 2% PL, 0.2% heparin (5,000 U/ml), and 1% antibiotic/antimycotic solution, and cultured under hypoxia (37°C, 1% O_2_), the combination of hypothermia and hypoxia (30°C, 1% O_2_), or control conditions (37°C, 20% O_2_) for 1, 4, and 8 days in α-MEM with supplements, with medium refreshment at day 4. At day 1, 4, and 8, cells were lysed for total RNA isolation, and quantification of total DNA, protein content, and alkaline phosphatase (ALP) activity as described below. We have chosen our time points based on earlier findings that hASCs show changes in proliferation, gene expression of KI67, COL1, and osteocalcin, and changes in ALP activity at 48 h, 4 days [26,27]. Moreover, since platelet lysate is a strong inducer of osteogenic differentiation in hASCs, we obtained statistical significant differences already at relatively early time points.

### Culture under hypoxia

For culturing in hypoxia, hASCs were placed in a NAPCO^®^ incubator (serial number 7101-C1, Precision Scientific Inc., Chicago, IL), in which the oxygen concentration is controlled by flushing with N_2_. Oxygen levels in the incubator were monitored by an internal oxygen sensor, as well as by external calibration using Dräger Tubes 6728081 (Drägerwerk Ag, Lübeck, Germany). Hypoxia was defined as 1% O_2_/5% CO_2_ in air.

### RNA isolation and gene expression analysis

Cells were washed with PBS and lysed with 700 μl TRIzol^®^ reagent (Life Technologies, Carlsbad, CA). Total RNA was isolated according to the manufacturer’s instructions. cDNA synthesis was performed using 750 ng of total RNA with reaction mixture Transcriptor First Strand cDNA synthesis kit (Roche Diagnostics, Mannheim, Germany), in an Applied Biosystems^®^ GeneAmp^®^ PCR System 9700, creating 20 μl suspension.

Real-time polymerase chain reaction (PCR) was used to determine gene expression of the osteogenic markers osteocalcin, collagen type 1 (*COL1*), and the chondrogenic marker *SOX9*. *KI67* gene expression was measured as proliferation marker. The ratio of *BAX*-to-*BCL-2* gene expression was used as an indicator of cell apoptosis. *VEGF-165* gene expression was measured as a marker of vasculogenesis. Three housekeeping genes (*TBP*, *HPRT*, and *YWHAZ*; InVitrogen, Carlsbad, CA) were used to correct for the combined effect of hypothermia and hypoxia. Real-time PCR reactions were performed with 1 μl cDNA (5x dilution) and ready to use hot start master mix LightCycler^®^ 480 SYBR Green I Master (Roche Diagnostics) in a LightCycler^®^ 480 Real-Time PCR System (Roche Diagnostics). The primer sequences are listed in Table 1.

**Table 1:** Primers used in the real-time PCR assay.

Gene	Oligonucleotide sequence		Accession no. genebank	An. temp (°C)	Amp. length (bp)
**Housekeeping genes**					
*TBP*	Forward	5' GGTCTGGGAAAATGGTGTGC 3'	NM_003194.4	56	97
	Reverse	5' GCTGGAAAACCCAACTTCTG 3'			
*HPRT*	Forward	5' GCTGACCTGCTGGATTACAT 3'	NM_000194	56	260
	Reverse	5' CTTGCGACCTTGACCATCT 3'			
*YWHAZ*	Forward	5' GATGAAGCCATTGCTGAACTTG 3'	NM_003406	56	229
	Reverse	5' CTATTTGTGGGACAGCATGGA 3'			
**Genes of Interest**					
*KI67*	Forward	5’ CCCTCAGCAAGCCTGAGAA 3’	NM_002417.4	56	202
	Reverse	5’ AGAGGCGTATTAGGAGGCAAG 3’			
Osteocalcin	Forward	5’ AGCCACCGAGACACCATGAGA 3’	NM_199173.5	57	288
	Reverse	5’ CTCCTGAAAGCCGATGTGGTC 3’			
*SOX9*	Forward	5’ CCACACTCCTCCTCCGGCATGA 3’	NM_000346	57	188
	Reverse	5’ TCCACGTCGCGGAAGTCGAT 3’			
*VEGF-165*	Forward	5' ATCTTCAAGCCATCCTGTGTGC 3'	NM_001025368.2	56	224
	Reverse	5' CAAGGCCCACAGGGATTTTC 3'			
*BAX*	Forward	5' CACCAGCTCTGAGCAGATCAT 3'	NM_138761.3	56	345
	Reverse	5' CTTGGTGCACAGGGCCTTG 3'			
*BCL-2*	Forward	5' GACTTCGCCGAGATGTCCAG 3'	NM_000633	56	232
	Reverse	5' AGGTGCCGGTTCAGGTACTC 3'			
*COL1*	Forward	5' TCCGGCTCCTGCTCCTCTTA 3'	NM_000088	56	336
	Reverse	5' GGCCAGTGTCTCCCTTG 3'			

An. temp = annealing temperature; amp. = amplicon; TBP = TATA-box binding protein; HPRT = Homo sapiens hypoxanthine phosphoribosyltransferase 1; YWHAZ = tyrosine 3-monooxygenase/tryptophan 5-monooxygenase activation protein zeta; *SOX9* = SRY-box 9; *VEGF-165* = Vascular endothelial growth factor; *BAX* = BCL2-associated X protein; *BCL-2* = B-cell CLL/lymphoma 2; *COL1* = collagen type 1.

### Total DNA, protein content, and ALP activity

hASCs were lysed with 0.7 ml of ice-cold Milli-Q water, harvested on ice, sonicated for 10 min in ice-cold water, and centrifuged for 10 min at 2,000 rpm at 4°C. The supernatants were immediately analysed for total DNA, protein content, VEGF protein concentration (see below), and ALP activity. Total DNA was quantified using the CyQUANT^®^ Cell Proliferation Assay (Molecular Probes, Eugene, OR) according to the manufacturer’s protocol. ALP activity was measured in the supernatant according to the method described by Lowry [28]. Total protein was determined using a BCA Protein Assay Reagent kit (Pierce, Rockford, IL). ALP activity and protein content were normalized for DNA. Absorbance was measured with a SynergyTM HT (BioTek Instruments, Winooski, VT) microplate reader in concordance with the manufacturer’s instructions.

### Nitric oxide

NO production was measured as nitrite (NO_2_^-^) accumulation in the conditioned medium (CM) using Griess reagent containing 1% sulfanilamide, 0.1% naphtylethelene-diamine-dihydrochloride, and 2.5 M H_3_PO_4_. Serial dilutions of NaNO_2_ in non-CM were used as a standard curve. Measurements were performed with a SynergyTM HT microplate reader.

### VEGF protein

To determine VEGF protein concentration in the supernatant, a Quantikine^®^ ELISA kit (R&D Systems Inc., Minneapolis, MN) was used and the samples were assayed according to the manufacturer’s protocol. Measurements were performed with a SynergyTM HT microplate reader.

### Statistical analysis

All data were checked for normality by using the Kolmogorov Smirnoff’s test. If applicable, logarithmic transformation was performed to obtain normal distributions. Mixed Model Analysis included temperature and oxygen concentration as fixed factors. The interaction between the factors was also evaluated. Statistical differences were considered significant if *p*<0.05. Post hoc multiple pairwise comparisons were performed at each time point with an adjusted significance level of 0.017 using Bonferroni’s method. All data were evaluated using the IBM SPSS statistical package for Macintosh, Version 20.0 (Armonk, NY).

## Results

**Fig 1:**
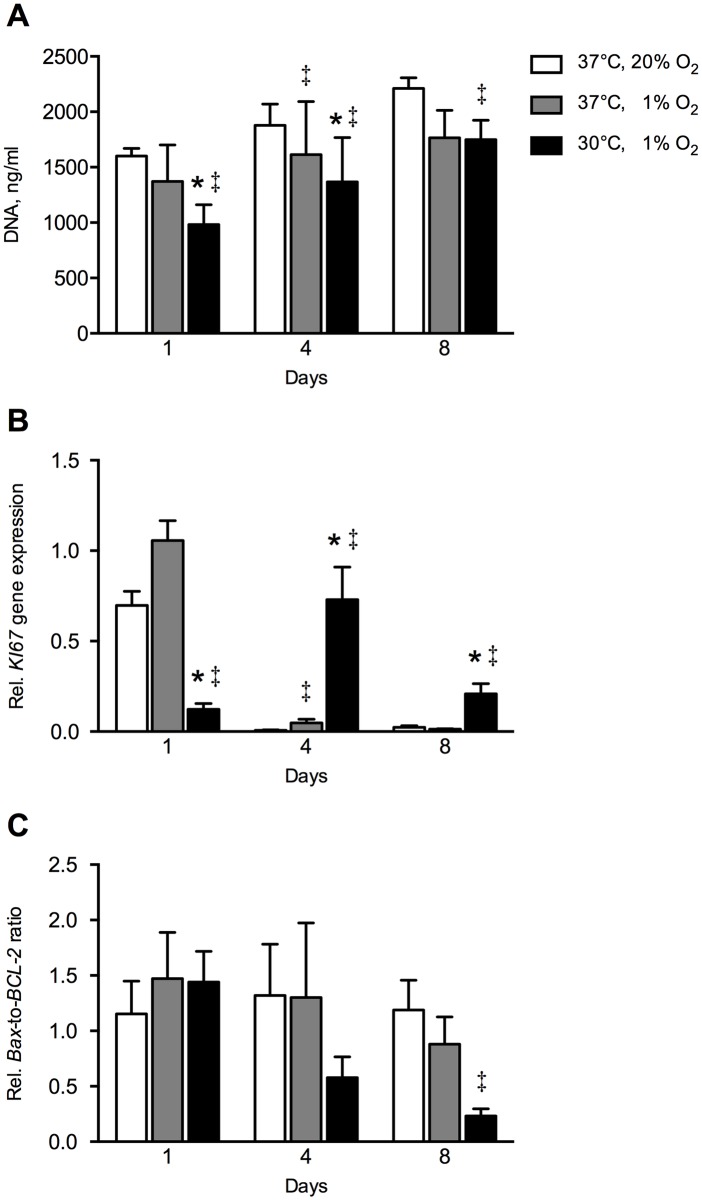
Effect of hypothermia and/or hypoxia on total DNA, *KI67* gene expression, and *BAX*-to-*BCL-2* gene expression ratio. (**A**) Total DNA. The combination hypothermia and hypoxia decreased total DNA at day 1 by 1.4-fold and at day 4 by 1.2-fold, but not at day 8 under hypoxia. **(B)** *KI67* gene expression. Hypoxia upregulated *KI67* gene expression at day 4 by 5-fold compared to controls. The combination hypothermia and hypoxia downregulated *KI67* gene expression at day 1 by 8.8-fold, but increased *KI67* gene expression at day 4 by 15-fold, and at day 8 by 21-fold under hypoxia. **(C)** *BAX*-to-*BCL-2* gene expression ratio. The combination hypothermia and hypoxia did not affect *BAX*-to-*BCL-2* ratio under hypoxia. The combination hypothermia and hypoxia decreased *BAX*-to-*BCL-2* ratio at day 8 by 5.1-fold compared to controls. Values are mean ± SEM, n = 10 from 3 independent experiments using ASCs obtained from 5 stem cell donors. *Significant effect of the combination hypothermia and hypoxia compared to hypoxia alone; ^‡^Significant effect compared to controls, *p*<0.05. Controls: 37°C, 20% O_2_. Gene expression is expressed relative to the average expression of the three housekeeping genes.

Hypoxia decreased total DNA at day 4 by 1.2-fold (*p* = 0.004) compared to controls (Fig 1A). The combination of hypothermia and hypoxia further reduced total DNA in hASCs at day 1 by 1.4-fold (*p* = 0.008), and at day 4 by 1.2-fold (*p* = 0.013), but not at day 8, compared to hypoxia alone.

Hypoxia upregulated *KI67* gene expression at day 4 by 5-fold (*p* = 0.0051) compared to controls (Fig 1B). The combination of hypothermia and hypoxia downregulated *KI67* gene expression at day 1 by 8.8-fold (*p* = 0.0007), but increased *KI67* gene expression at day 4 by 15-fold (*p* = 0.001) and at day 8 by 21-fold (*p* = 0.002), compared to hypoxia alone (Fig 1B). Hypoxia did not significantly affect *BAX*-to-*BCL-2* gene expression ratio. The combination of hypothermia and hypoxia decreased the *BAX*-to-*BCL-2* gene expression ratio at day 8 by 5.1-fold (*p* = 0.016) compared to controls, but not compared to hypoxic conditions (Fig 1C).

**Fig 2:**
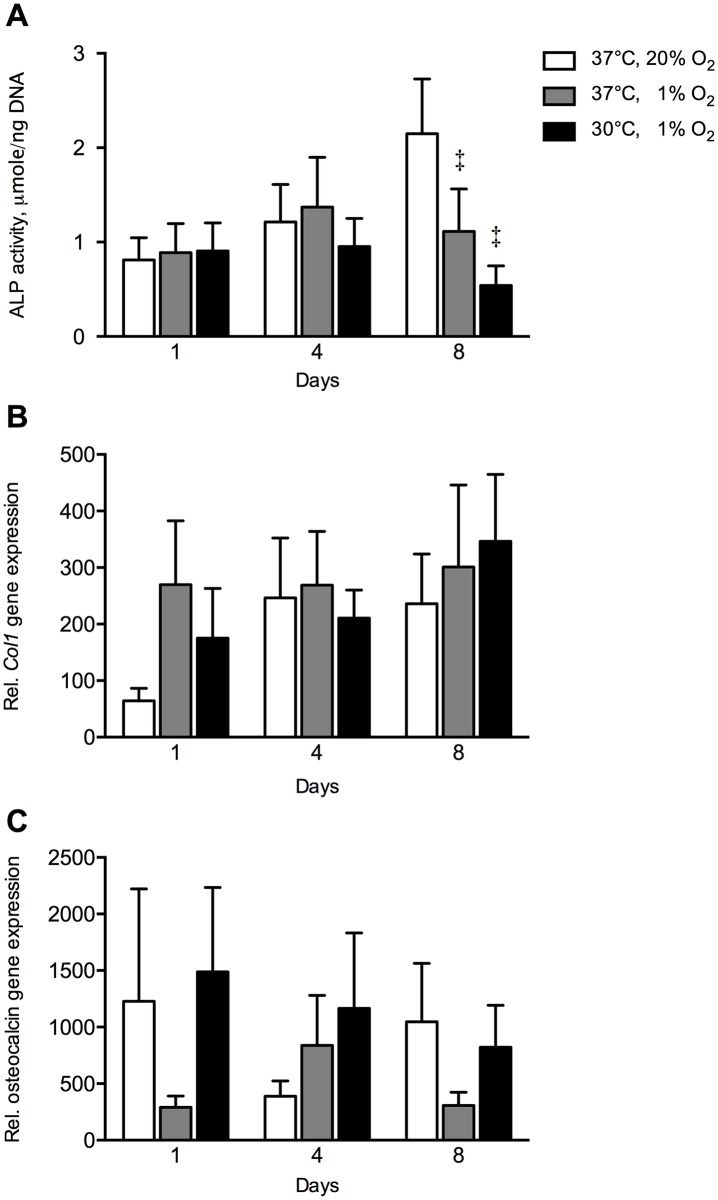
Effect of hypothermia and/or hypoxia on ALP activity, *COL1*, and osteocalcin gene expression. **(A)** ALP activity normalized for cell number. The combination hypothermia and hypoxia did not affect ALP activity under hypoxia. Hypoxia decreased ALP activity at day 8 by 4-fold compared to controls. **(B)** *COL1* gene expression. Hypoxia and the combination hypothermia and hypoxia did not affect *COL1* gene expression. **(C)** Osteocalcin gene expression. Hypoxia and the combination hypothermia and hypoxia did not affect osteocalcin gene expression. Values are mean ± SEM, from n = 10 of 3 independent experiments using ASCs obtained from 5 stem cell donors *Significant effect of the combination hypothermia and hypoxia compared to hypoxia alone; ^‡^Significant effect compared to controls, *p*<0.05. Controls: 37°C, 20% O_2_. Gene expression is expressed relative to the average expression of the three housekeeping genes.

Hypoxia reduced cell-associated ALP activity, a marker of osteogenic differentiation, at day 8 by 1.9-fold (*p* = 0.015) compared to control hASCs (Fig 2A). The combination of hypothermia and hypoxia reduced cell-associated ALP activity at day 8 by 4-fold (*p* = 0.016) compared to control MSCs, but no effect of hypothermia under hypoxia was found compared to hypoxic conditions. Hypoxia nor the combination of hypothermia and hypoxia affected the gene expression of osteogenic markers *COL1* and osteocalcin (Fig 2B and 2C).

**Fig 3:**
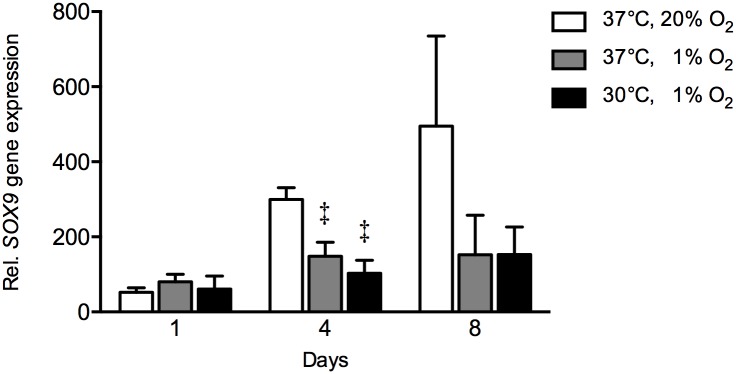
Effect of hypothermia and/or hypoxia on *SOX9* gene expression. The combination hypothermia and hypoxia did not affect *SOX9* gene expression under hypoxia. Hypoxia reduced *SOX9* gene expression at day 4 by 2.9-fold compared to controls. Values are mean ± SEM, from n = 12 of 3 independent experiments using ASCs obtained from 6 stem cell donors. Gene expression is expressed relative to the average of the three housekeeping genes. ^‡^Significant effect compared to controls, *p*<0.05. Controls: 37°C, 20% O_2_.

Hypoxia alone reduced gene expression of the chondrogenic marker *SOX9* at day 4 by 2-fold (*p* = 0.006) compared to controls in hASCs (Fig 3), while the combination of hypothermia and hypoxia reduced *SOX9* gene expression at day 4 by 2.9-fold (*p* = 0.006) compared to controls. As a result, the combination of hypothermia and hypoxia did not affect *SOX9* gene expression compared to hypoxic hASCs.

**Fig 4:**
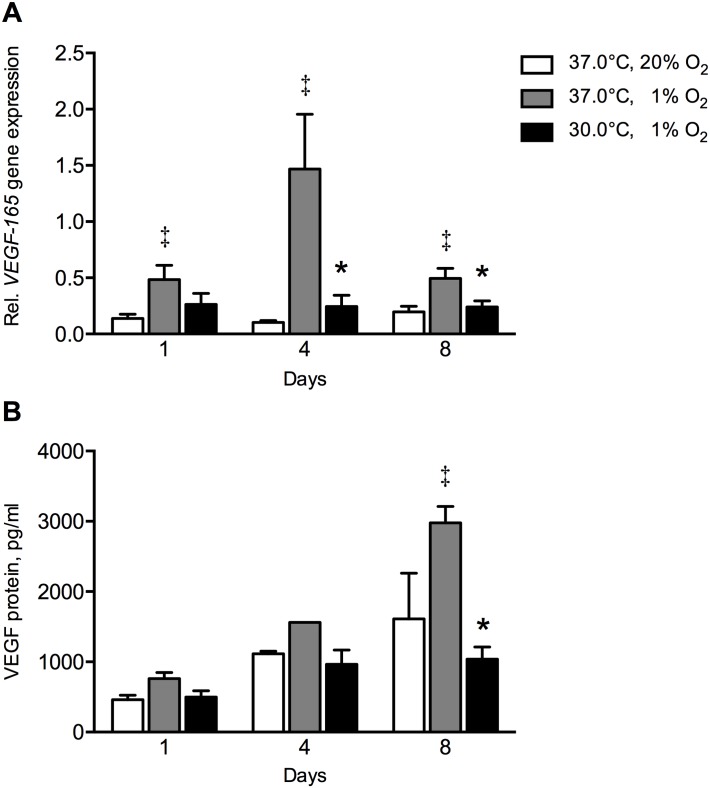
Effect of hypothermia and/or hypoxia on VEGF gene and protein expression. (**A**) *VEGF-165* gene expression. The combination hypothermia and hypoxia decreased *VEGF-165* gene expression at day 4 by 6-fold and at day 8 by 2.1-fold under hypoxia. Hypoxia upregulated *VEGF-165* gene expression at day 1 by 3.5-fold, at day 4 by 15-fold, and at day 8 by 2.5-fold compared to controls. (**B**) VEGF protein expression. The combination hypothermia and hypoxia decreased VEGF protein concentration by 2.9-fold compared to hypoxia alone at day 8. Hypoxia increased VEGF protein concentration by 1.9-fold compared to controls at day 8. Values are mean ± SEM, from n = 12 of 3 independent experiments using ASCs obtained from 6 stem cell donors for *VEGF-165* gene expression, and n = 7 of 3 independent experiments from 4 stem cell donors for VEGF protein concentration. Gene expression is expressed relative to the average expression of the three housekeeping genes. *Significant effect of the combination hypothermia and hypoxia compared to hypoxia alone; ^‡^Significant effect compared to controls, *p*<0.05. Controls: 37°C, 20% O_2_.

Hypoxia upregulated *VEGF-165* gene expression at day 1 by 3.5-fold (*p* = 0.008), at day 4 by 15-fold (*p* = 0.0005), and at day 8 by 2.5-fold (*p* = 0.007), compared to controls (Fig 4A). The combination of hypothermia and hypoxia decreased *VEGF-165* gene expression at day 4 by 6-fold (*p* = 0.007) and at day 8 by 2.1-fold (*p* = 0.002), compared to hypoxia alone (Fig 4A).

Hypoxia increased VEGF protein concentration at day 8 by 2.9-fold (*p* = 0.003) compared to controls (Fig 4B). The combination hypothermia and hypoxia decreased VEGF protein concentration at day 8 by 1.9-fold (*p* = 0.0011) compared to hypoxia (Fig 4B).

**Fig 5:**
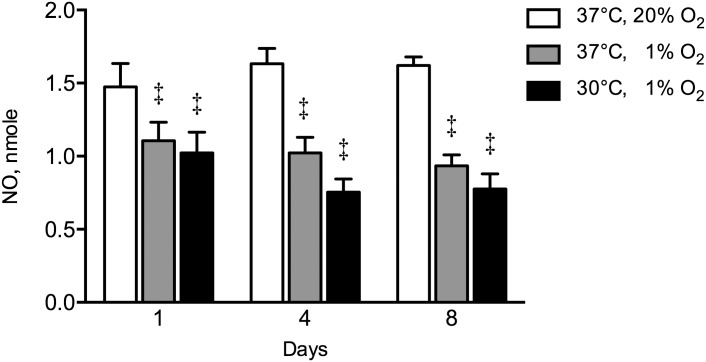
Effect of hypothermia and/or hypoxia on NO production. The combination hypothermia and hypoxia did not affect NO production under hypoxia. Hypoxia decreased NO production at day 1 by 1.3-fold, at day 4 by 1.9-fold, and at day 8 by 1.7-fold compared to controls. Values are mean ± SEM, from n = 12 of 3 independent experiments using ASCs obtained from 6 stem cell donors. ^‡^Significant effect compared to controls, *p*<0.05. Controls: 37°C, 20% O_2_.

Hypoxia reduced NO production at day 1 by 1.3-fold (*p* = 0.006), at day 4 by 1.9-fold (*p* = 0.007), and at day 8 by 1.7-fold (*p* = 0.011), compared to controls (Fig 5). The combination of hypothermia and hypoxia reduced NO production at day 1 by 1.4-fold (*p* = 0.0009), at day 4 by 2.2-fold (*p* = 0.017), and at day 8 by 2.1-fold (*p* = 0.004), compared to controls. As a result, NO production by hASCs under the combination of hypothermia and hypoxia was comparable to that by cells under hypoxia alone (Fig 5).

**Fig 6:**
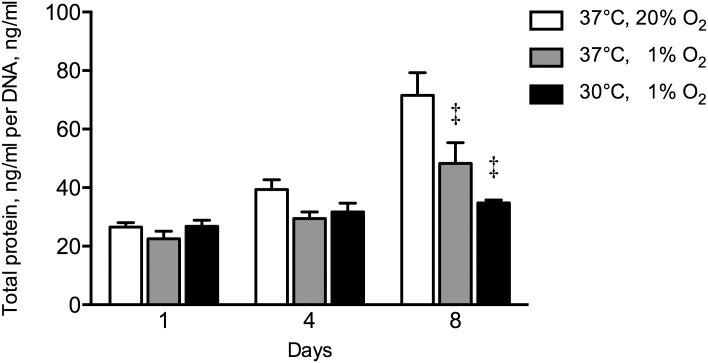
Effect of hypothermia and/or hypoxia on total protein content normalized for cell number. The combination hypothermia and hypoxia did not affect total protein under hypoxia. Hypoxia decreased total protein at day 8 by 1.5-fold compared to controls. Values are mean ± SEM, from n = 12 of 3 independent experiments using ASCs obtained from 6 stem cell donors. ^‡^Significant effect compared to controls, *p*<0.05. Controls: 37°C, 20% O_2_.

Hypoxia reduced total protein production rate at day 8 by 1.5-fold (*p* = 0.002), compared to control hASCs (Fig 6). The combination of hypothermia and hypoxia similarly reduced total protein production rate at day 8 by 2.1-fold (*p* = 0.001) compared to controls.

## Discussion

Hypothermia is used in fracture patients to enhance recovery [4]. It has been suggested that this enhanced recovery originates from modulation of the inflammatory reaction [2,3]. Hypothermia may also modulate the effects of ischaemia and hypoxic conditions that are prevalent in the wound environment, but whether the combined effect of hypothermia and hypoxia is beneficial for the cells responsible for callus formation is unknown.

The hypoxic environment in which osteoprogenitor cells naturally reside is thought to stimulate proliferation [29]. We found that in our culture conditions hypoxia actually inhibits MSC number. We hypothesized that hypothermia attenuates this hypoxia-induced inhibition of MSC number. We found that the combination of hypothermia and hypoxia reduced cell number and *KI67* gene expression compared to hypoxic hASCs already after 1 day of culture, but it stimulated *KI67* gene expression at day 4, probably leading to the observed catch-up effect in cell number after 8 days. This catch-up effect combined with unchanged apoptosis suggests increased proliferation of hASCs after hypothermia treatment of hypoxic MSCs. Hypothermia (35.5°C) has been reported to transiently reduce the number of osteoblasts cultured under normoxia [21]. It initially reduces cell number by 20% after 4 days of culture under normoxia and recovers to control levels after 7 days [21]. Under normoxia, hypothermia (33°C) was also reported to reduce DNA synthesis by 24% in bone marrow MSCs after 4 days of culture compared to normothermic MSCs [30]. We found that hypoxia reduced hASC proliferation at 37°C, which agrees with published data showing reduced proliferation of human bone marrow MSCs cultured under hypoxic conditions (1% O_2_) [31]. Apparently, the effect of hypothermia on cell proliferation differs under hypoxic and normoxic conditions.

Hypothermia regulates the expression of various genes involved in necrotic and apoptotic pathways, such as the PI3K/Akt pathway, but the mechanisms have not been completely elucidated to date [32]. Hypothermia attenuates the increase in Bax gene expression associated with ischaemic brain damage in a rodent model [33]. Consequently, the pro-apoptotic effect of Bax protein on mitochondrial membrane potential is reduced by hypothermia, which may cause an anti-apoptotic effect [33]. We questioned whether apoptosis is stimulated by hypoxia, and whether hypothermia attenuates the stimulation of apoptosis in hypoxic MSCs. We found that apoptosis (assessed as *BAX*-to-*BCL-2* gene expression ratio) was not affected by hypoxia, nor did hypothermia modulate cell apoptosis under hypoxic conditions in hASCs.

We determined whether osteogenic differentiation is increased in hypoxia, and if hypothermia attenuates the hypoxia-induced stimulation of hASCs. Diverging data exist about the effect of hypoxia on MSC differentiation [31,34–36]. Bone marrow-derived human MSCs cultured under a 2% oxygen tension for one month exhibit increased expression of early osteogenic differentiation markers osteonectin and alkaline phosphatase activity compared to cells grown under normoxic conditions [34]. A murine MSC line C3H/10T1/2 pre-incubated with the hypoxia mimicking agent CoCl_2_ for 24 h shows enhanced osteogenic differentiation and mineralization [35]. In contrast, *in vitro* osteogenesis and chondrogensis is severely diminished in hASCs after 3 weeks of culture under a 2% oxygen tension [36]. A decrease in ALP activity in combination with increased expression of late markers for osteogenesis have been shown in hypoxic (1% O_2_) human bone marrow stem cell cultures [31]. This suggests that a hypoxic environment might alter the timing of sequential gene expression in the osteogenic differentiation process, and consequently early osteogenic differentiation markers are reduced compared to late markers [31]. This is in partial accordance with our results, as we showed that hypoxia significantly reduced cell-associated ALP activity, but did not affect *COL1* and osteocalcin compared to control hASCs. It is likely that the timing and degree of hypoxia influences MSC differentiation. Enhanced expression of late differentiation markers might be possible at later time points than day 8.

Under normoxia, hypothermia (35.5°C) has been shown to reduce ALP activity, osteocalcin, and Col1 gene expression, and to decrease bone formation by 70% in murine osteoblasts [21]. The combination of hypothermia and hypoxia in our experiments might thus be expected to result in a stronger inhibition of MSC differentiation than hypoxia alone. In our study with hASCs we found that, under hypoxia, hypothermia did not affect cell-associated ALP activity, *COL1* gene expression, nor osteocalcin expression in hASCs.

We found that hypoxia reduced gene expression of the chondrogenic marker *SOX9* after 4 days of culture compared to controls. *SOX9* gene expression is significantly upregulated in human bone marrow stem cells cultured under 1% O_2_ compared to normoxia after 14 days [31]. Moreover, human embryonic stem cells cultured under hypoxia (5% O_2_) show increased *SOX9* gene expression after 14 days [37]. Murine C3H10/T1/2 cells pre-incubated with a hypoxia mimicking agent, show increased Sox9 gene expression after 3 days of culture [35]. Hypoxia (3% O_2_) has also been shown to enhance chondrogenesis in ovine bone marrow MSCs cultured on porous scaffolds for 14 days compared to normoxia [29]. Please note that under normal physiological conditions, human bone marrow cells reside under 6% O_2_ [38]. When cells reside under 3–5% O_2_ this might not be considered as a severe hypoxic condition (as in our study) [38]. This could explain the difference between our results and the results reported in literature. In addition, our 2-dimensional culture conditions do not naturally favour chondrogenic differentiation.

We expected an upregulation of *SOX9* gene expression under hypoxia since chondrogenesis is driven by HIF-1α [39], and HIF-1α is upregulated under hypoxia [1]. HIF-1α also affects VEGF [12,39] and we showed a significant upregulation of *VEGF-165* gene expression under hypoxia after 4 days of culture, even though we were unable to demonstrate an effect of hypoxia on *SOX9* gene expression. Hypothermia significantly reduced *VEGF-165* gene expression, as well as VEGF protein expression under hypoxia, yet we were unable to demonstrate an effect of hypothermia on *SOX9* gene expression under hypoxia in hASCs. Further analysis on HIF-1α might elucidate these intriguing results.

We found that *VEGF-165* gene expression and VEGF protein expression were reduced by hypothermia under hypoxia. This is in accordance with another study showing that VEGF production decreases by 30% in hypoxic (1% O_2_) retinal pigment epithelial (ARPE-19) cells exposed to moderate (34°C) hypothermia [40]. VEGF is a bone-metabolism cytokine that stimulates the proliferation and chemotactic migration of osteoblast precursor cells [11]. Thus reduced VEGF levels may decrease osteoblast proliferation and possibly also differentiation, yet we did not find a reduction in ALP activity or osteocalcin gene expression as a result of hypothermia in our hypoxic culture conditions. This suggests that the early stages of fracture healing are not adversely affected by hypothermia under hypoxic conditions. Being a powerful inducer of angiogenesis, VEGF is important for the later stages of bone healing when vascularization of the callus is warranted [41]. Hence reduced VEGF levels as a result of hypothermia might have implications for the later stages of bone healing.

Since hypoxia increases oxidative stress, we hypothesized that hypoxia stimulates NO production, and that hypothermia attenuates the hypoxia-induced increase in NO production of MSCs. NO production was consistently reduced under hypoxia compared to control hASCs. Hypothermia did not affect NO production by hASCs under hypoxia. In physiological conditions, a low NO level maintains vasculature tone [42]. In pathological conditions, such as hypoxia in ischaemic-reperfusion injury, NO levels will ultimately increase by the activity of inducible NO synthase (iNOS) after reperfusion, thereby contributing to overall oxidative stress [43,44]. NO production requires oxygen since it is synthesized from L-arginine and O_2_. In our experiments, there is no increase in oxygen tension as occurs during reperfusion after ischaemia. This might explain the lack of an increase in NO production by hASCs cultured under hypothermia. Mast cells likely play a key role in iNOS-mediated augmentation of oxidative stress, and lack of these cells might not lead to a full-blown cascade with increased NO concentrations [44].

Some care must be taken when interpreting our results. We used hASCs since these cells can differentiate along the osteogenic lineage, they can be stimulated by hypoxic conditions and are readily available, and thereby resemble periosteum-derived osteoprogenitor cells responsible for bone repair *in vivo* [8,45–47]. The frequency of BMSCs in human bone marrow is low, and proliferative and differentiation capacity of BMSC is partially lost during cell expansion [48]. However, in contrast to bone marrow, adipose tissue contains a high stem cell to volume ratio [49], and it can be processed within a short time frame to obtain highly enriched ASC preparations. ASCs show many similarities with BMSCs with regard to surface marker profiles, multi-lineage potential, and growth properties [50,51]. The hASCs used in our study have been characterized previously by our group [22,23,51]. Our *in vitro* data provide insight in the mechanism of the effect of cryotherapy on MSCs, but *in vivo* experiments are needed to draw firm conclusions about the effect of cryotherapy on bone repair. Note that we tested the effect of hypothermia in cells under hypoxia, but not normoxia, since this was beyond the scope of our study, where we aimed to mimic the hypoxic fracture environment. Cell culture was performed under ambient oxygen levels, and therefore the cells were exposed to acute hypoxia during the course of the experiments. The naturally occurring stem cell niche in which most stem cells grow or reside is a hypoxic environment, and therefore our control condition is in fact hyperoxic and might alter hASC characteristics [6]. Most studies cited [3,18,19,21,30,33] address hypothermia at near physiological temperature (33–37°C). However cryotherapy causes hypothermia at a lower temperature, i.e. outside the physiological range (<33°C). Cryotherapy lowers the temperature to 23°C in healthy individuals at 1.5 cm below the subcutaneous fat layer [15]. Currently no reports are available that show temperature decline at the bony level after induced hypothermia treatment in fracture patients. One study measuring the temperature decline found that after induced hypothermia at 1 cm (23.5°C) and 2 cm (26.4°C) below the subcutaneous fat layer found that temperature decline during induced hypothermia and tissue depth are inversely related [52]. Based on an estimated distance between the subcutaneous fat layer and the bone (~3 cm), and the inverse relationship between temperature decline by hypothermia and tissue depth we estimated the temperature at a deeper bony level of the thigh to be 30°C. We applied intra-hypoxic hypothermia, i.e. hypothermia commenced and continued at the same time and as long as hypoxia treatment. Hence care should be taken when translating these *in vitro* results to an *in vivo* situation, since clinically hypoxia or ischaemia is usually present before starting hypothermia treatment, and hypothermia treatment might not be applied continuously.

In conclusion, our data show that under hypoxia, hypothermia reduced *VEGF-165* gene expression and VEGF protein expression, but did not affect cell number, nor osteogenic or chondrogenic differentiation, or NO production by hASCs. Decreased VEGF gene and protein expression might ultimately reduce vasculogenesis, which may impair later stages of bone healing *in vivo*.

## Supporting information

S1 File Study data. (XLSX)
